# The Internet: Friend or Foe of Antibiotic Resistance? Results of a Cross-Sectional Study among Italian University Students

**DOI:** 10.3390/antibiotics10091091

**Published:** 2021-09-09

**Authors:** Francesca Licata, Silvia Angelillo, Alessandra Oliverio, Aida Bianco

**Affiliations:** Department of Health Sciences, School of Medicine, University of Catanzaro “Magna Græcia”, Viale Europa, 88100 Catanzaro, Italy; francesca.licata@studenti.unicz.it (F.L.); silvia.angelillo@studenti.unicz.it (S.A.); alessandra.oliverio@studenti.unicz.it (A.O.)

**Keywords:** antibiotics, antibiotic resistance, internet, university students, Italy

## Abstract

The study aims were to investigate knowledge and attitudes towards antibiotics and antibiotic resistance (ABR), and to assess the extent of practices regarding antibiotic consumption and Internet use among university students in Southern Italy. Data were collected through an anonymous online questionnaire from 1 April to 14 April 2021. The eligibility criteria for the study were: (i) age between 18 and 30 years and (ii) registered as an undergraduate student at the university. Among the 1051 sampled students, only 7.4% gave the correct answer to all 7 knowledge statements about antibiotics and ABR. The main determinants of knowledge were being younger and attending to medical or life sciences majors. Almost two-thirds took an antibiotic in the previous 12 months and 24.6% reported having self-medicated with antibiotics. More than half of the sample used the Internet to seek information about antibiotics and/or ABR, and it was the strongest predictor of self-medication with antibiotics. The study findings highlighted gaps in knowledge, considerable antibiotic consumption, sometimes without prescription, together with an extensive Internet use to seek health-related information. To facilitate the health-promoting use of the Internet in conjunction with health care providers, we could make young adults aware of the rational use of antibiotics.

## 1. Introduction

Antibiotics have proven to be invaluable in treating infections that arise in everyday life and also in the prevention of infections during periods when patients may be particularly vulnerable. However, the use of antibiotics is leading to ever increasing numbers of antibiotic-resistant bacteria worldwide, leading to a reduction in the effectiveness of antibiotic treatments [1]. In addition, the lack of new drugs to replace old ones probably makes antibiotic resistance (ABR) one of the greatest human health and sustainability challenges of the 21st century [2].

The current body of evidence indicates that among the factors that have led to an existing crisis on antibiotics worldwide is overuse in community settings [3,4] that requires effective interventions including changing public awareness and subsequent antimicrobial stewardship (AMS) behaviors [5]. Although AMS is most commonly thought of in clinical settings, the word “stewardship” means “taking care of”, particularly on behalf of others, and the collaborative effort of all stakeholders is decisive to minimize the global problem of ABR [6]. In particular, the public’s behaviors, such as those related to adhering to physicians’ prescriptions, purchasing antibiotics without prescription, and safe disposal of leftover antimicrobials represent central factors in reducing the drivers of ABR [7].

Among the public, students are “open” to new information, since learning is their job. Students are at the stage of learning where their knowledge and attitudes are amenable to modification. This is a good starting point to introduce concepts such as the rational use of drugs [8]. Previously published studies showed that university students needed more education on the appropriate use of antibiotics [9], and that a greater emphasis on infectious disease education is also required by the future medical workforce [10]. Future physicians and pharmacists have to be prepared to rationally manage the use of antibiotics and to promote patient awareness and knowledge regarding their rational use [11,12,13,14,15,16]. Improving awareness of ABR through communication, education, and training was introduced as one of the five objectives of a global action plan on antimicrobial resistance by the World Health Organization (WHO), Geneva, Switzerland [3].

Especially among young adults, the Internet offers a simple, inexpensive, and highly accessible means of communication. The Internet is frequently used to search for information on antibiotics, as reported by a recent study that found that Google users’ interest in antibiotics for the years 2004–2019 has increased significantly [17]. Some authors suggest including social media as a tool for health-related education in this area [18]. However, information spread via the Internet may be less subject to fact-checking and verification than traditional mass media outlets (i.e., radio, television, and newspaper). In addition, ABR could be exacerbated by patients self-medicating with antibiotics from online pharmacies. Therefore, it is vital that governments and regulators clamp down on unlicensed Internet sales of antibiotics. As the WHO highlighted the global action plan for tackling ABR [3], strengthening international regulations on the distribution and use of antibiotics, with emphasis placed on those obtained through Internet sales, is strongly needed [3]. In Italy, for online pharmacies to operate legally, they must be licensed by the Ministry of Health and the sale and dispensation of pharmaceutical products that require a prescription were prohibited by law [19].

The aims of the present study were to investigate the level of knowledge and attitudes towards antibiotics and ABR, and to assess the extent of practices regarding antibiotic consumption and Internet use among undergraduate university students in Southern Italy. A secondary objective was to test the relationship between the use of the Internet to seek health information and overuse of antibiotics.

## 2. Results

### 2.1. Practices Regarding Antibiotic Consumption and Internet Use

Table 1 shows practices regarding antibiotic consumption and Internet use. Nearly all subjects (97.2%) had taken antibiotics at least once in their life and, among these, 62.6% had done so in the last year. The vast majority of the sample (80%) reported keeping leftover antibiotics for future use, and just 5.6% returned unused antibiotics to the community pharmacies.

The vast majority of participants (i.e., 95.4% from social sciences, 94.5% from medical or life sciences, and 94.1% from technology majors) used the Internet to seek health-related information, and 19.4% of them sought health information daily. The most reported reasons for using the Internet to seek health information were to learn more about diseases (88.9%) and/or therapies (57.4%). Interestingly, 66.7% of the sample sought information on antibiotics and/or ABR from the Internet, and among those 73.2% from medical or life sciences, 61.5% from technology and 54.6% from social sciences majors. Less than one-fifth (17.1%) of the sample bought pharmaceutical products online at least once during their lifetime, and the most common reasons were because it was considered cheaper (76.5%) and more convenient (50.2%) than buying them at the community pharmacy. Furthermore, just 4.5% of the sample reported having bought antibiotics online, and 12.5% of them claimed that they needed a prescription for the purchase. Almost a quarter of the students sampled (i.e., 24.9% of those from technology, 24.8% of those from social sciences, and 24.4% of those from medical or life sciences majors) reported having self-medicated with antibiotics and the most frequent reasons were lack of time to consult the physician (72.1%) and having used them before for a similar illness (25.1%). Among the students that self-medicated with antibiotics, only 17.4% had difficulty in getting an antibiotic from the pharmacist without a prescription. The results of the bivariate analysis showed that Internet use to seek information about antibiotics and/or ABR was significantly less frequent among students attending to social sciences [odds ratio (OR): 0.44; 95% confidence interval (CI): 0.33–0.60] or technology majors (OR: 0.59; 95% CI: 0.41–0.84) compared with those attending to medical or life sciences majors. No statistically significant differences were observed between practices regarding antibiotics consumption and Internet use and attending a specific major. Results of the logistic regression analysis showed that the use of the Internet to seek information about antibiotics and/or ABR (OR: 1.69; 95% CI: 1.20–2.38) and to buy pharmaceutical products online (OR: 1.95; 95 % CI: 1.36–2.78) were strong predictors of self-medication with antibiotics (Model 1 in Table 2). Moreover, each year of age (OR: 1.05; 95 % CI: 1.00–1.11) resulted in a 5% increase in the odds of having self-medicated with antibiotics. The logistic model provided satisfactory goodness of fit (*p*-values of 0.20). Those results were confirmed after removing the variables with a *p*-value > 0.05, except for age, which no longer predicted the outcome of interest.

### 2.2. Participant Demographics

Of the 1450 selected students invited to participate in the study, 1051 answered the questionnaire, giving a response rate of 72.5%. Among them, 69.2% were female and the mean age was 22 years [standard deviation (SD) ± 5.2]. Nearly all the participants were Italians (98.7%) whereas just 9.9% of the sample reported having a chronic disease. Of all participants, 59.1% were enrolled in medical or life sciences (i.e., medicine, nursing, pharmacy, dentistry, and other healthcare professions), almost a quarter (24.8%) in social sciences, and 16.2% in technology majors. Almost all of the participants (99.2%) used the Internet daily.

### 2.3. Knowledge about Antibiotics and ABR

Only 7.4% of the respondents gave the correct answer to all 7 knowledge statements, with an overall median knowledge score of 4 (IQR 4–6). The knowledge of the participants about antibiotics and ABR is reported in Table 3.

The results of the bivariate statistical analysis showed that attending to medical or life sciences majors was significantly associated with good knowledge. Specifically, compared with medical or life sciences students, those who attended to social sciences or technology majors were significantly less knowledgeable that antibiotics are not useful to treat viral infections (OR: 0.13; 95% CI: 0.09–0.18 and OR: 0.21; 95% CI: 0.15–0.31, respectively), that you can not stop taking the antibiotic when you start feeling better (OR: 0.47; 95% CI: 0.31–0.71 and OR: 0.48; 95% CI: 0.30–0.77, respectively), and that self-medication with antibiotics contributes to the spread of ABR (OR: 0.12; 95% CI: 0.08–0.17 and OR: 0.23; 95% CI: 0.15–0.34, respectively). Furthermore, attending to social sciences majors (OR: 0.20; 95% CI: 0.07–0.54) was negatively associated with knowledge that it is wrong to take antibiotics that individuals with the same symptoms have used without consulting the doctor, compared with students from medical or life sciences majors. The results of the ordinal regression model predicting knowledge about antibiotics and ABR showed that every year of age (Coeff: 0.09; 95% CI: 0.05–0.12) and attending to medical or life sciences majors (Coeff: 1.13; 95 % CI: 0.90–1.37) were positively associated with better knowledge. Instead, a lower level of knowledge was observed in females (Coeff: −0.25; 95% CI: −0.50–0.01) compared with males (Model 2 in Table 2). The statistically significant associations with the outcome of interest were confirmed in the model built after removing the variables with a *p*-value > 0.05. Similarly, the estimates of the model built removing the collinear variables were fully confirmed.

### 2.4. Attitudes towards ABR and Online Purchase of Pharmaceutical Products and Antibiotics

Almost two-fifths of the sample (37%) believed that ABR is a problem that could not be solved through the marketing of new antibiotics. The results of the multiple logistic regression analysis indicated that an increase of the knowledge score about antibiotics and ABR (OR: 1.21; 95% CI: 1.09–1.33) and each year of age (OR: 1.06; 95% CI: 1.02–1.11) resulted, respectively, in a 21% and 6% increase in believing ABR is a problem that could not be solved through the marketing of new antibiotics (Model 3 in Table 2). Moreover, attending to medical or life sciences majors (OR: 2.02; 95% CI: 1.52–2.69) and having a chronic disease (OR: 1.74; 95% CI: 1.14–2.65) were positively associated with that attitude. The logistic regression model provided satisfactory goodness of fit (*p*-values of 0.18). The results of the logistic regression model built after removing the variables with a *p*-value > 0.05 confirmed the findings reported above. Similarly, the estimates of the model built removing the collinear variables were fully confirmed. Regarding the online purchase of pharmaceutical products, 18.4% and 22.2% of the students believed that it is a safe and a positive possibility, respectively. Moreover, almost two-thirds of the sample (61.7%) judged the online purchase of antibiotics as a negative possibility due to an increased risk of receiving counterfeit substances.

## 3. Discussion

The present study has investigated the knowledge, attitudes, and practices regarding antibiotic consumption and ABR among university students, with a specific focus on the potential role of the Internet as a tool to tackle ABR. Antibiotic overuse and the development of ABR is a transboundary and global problem, and the findings of the present survey provide an up-to-date insight that will aid in the design of novel interventional strategies to promote prudent antibiotic use among young adults.

This survey has provided four major findings. First, the findings underlined gaps in knowledge about antibiotic use. The fact that more than one-third of the study population believed that antibiotics are effective for viral infections, demonstrates clear misconceptions and confusion regarding the correct indication for antibiotic use. From the ordinal logistic regression analysis, the main determinants of knowledge about antibiotics were age and majors, and suggests that, if there are limited resources, educational programs on antibiotics should be targeted at younger students from programs that are non-life sciences based. The finding that respondents from the medical or life sciences majors had significantly better knowledge than students from the other courses is in line with previous studies [20,21,22]. As expected, those students had more exposure to scientific knowledge related to antibiotics and ABR compared with students from other majors.

Second, a fairly high degree of antibiotic consumption was evident among participants in the study. Indeed, 62.6% of the respondents used antibiotics in the 12 months prior to the study, and this proportion is very similar to that of an earlier study in the same area [7], but the earlier study was conducted on a different target population, i.e., the general public. This implies that the use of antibiotics among university students can be considered quite high as this group of people are younger and hence, are presumably healthier than adult individuals in the earlier study. Indeed, a previous European study revealed that starting from 20–24 years old the average yearly antibiotic consumption gradually increases with individual age, with the highest prevalence rates values for over 80-year-old individuals [23]. Similar trends were reported by previously published Italian studies [24,25].

Thirdly, approximately one-quarter of the respondents had taken an antibiotic without a prescription, although the accessibility to antibiotics is strictly regulated and no antibiotics should be distributed without written prescription by a physician. Even though this proportion is lower than that reported in a previous Italian study [26], the fact that only a very small proportion (17.4%) of students that self-medicated with antibiotics had difficulty in getting antibiotics from the pharmacist highlights that it could be easy for individuals to misuse these drugs. In this context, community pharmacists could play a pivotal role. Indeed, it was demonstrated that about 4 out of 10 Italian community pharmacists had dispensed antibiotics without prescription [27], and that pharmacists’ knowledge regarding the overuse of antibiotics as the main cause of ABR was lacking [28]. An inadequate community pharmacist’s knowledge could be translated to a lack of information being disseminated to the public. In addition, a vast majority of the sample reported keeping leftover antibiotics for future use, a practice that is known to contribute to self-medication. Policy makers should also pay special attention to the regulations of leftover antibiotic use aimed at dispensing precise doses instead of whole packages of antibiotics [7].

The fourth key result is that searching for health and antibiotic related information online appeared extensive among university students in the sample. The Internet is broadly recognized as a potentially important instrument for transforming medical care and public health [29] The Internet could be promising as a health communication and education tool [30], and it could be a key resource in health behavior change interventions and programs. However, the finding that using the Internet to search for antibiotic-related information and to buy pharmaceutical products was a potential predictor of self-medication with antibiotics deserves attention. Moreover, it should be noted that the Internet could induce individuals to not adhere to the prescribed antibiotic dosing regimens. Possible dangers of self-medication, non-adherence to prescribed therapy, and inappropriate antibiotic use should be communicated to the general public by providing relevant and easy-to-understand information on the Internet [31]. Moreover, it has also been demonstrated that one of the contributing factors to the misconceptions about antibiotics may be unreliable sources of information, such as people who shared their own experiences on antibiotic use through social media [32].

To appreciate the findings of this cross-sectional study, some potential limitations need to be considered. First, self-reporting of information increases the occurrence of bias. Intentional deception, poor recall, or misunderstanding of the questions can all contribute to the wrong assumption of actual practices regarding antibiotic consumption. Nevertheless, it was demonstrated that the means for improving the validity of self-reported data should include adherence to procedures that maximize anonymity and confidentiality, as we performed in the survey. A second limitation of this study pertained to the cross-sectional design not allowing us to draw conclusions on causality about the observed associations and to the self-reporting of practices. Third, our study involved university students in one region of Italy, and the results cannot be generalized to young adults in the whole country. However, we are confident that the findings of the study may be representative at least for the university students of the regions of southern Italy.

## 4. Materials and Methods

### 4.1. Study Design

This cross-sectional study was conducted over a two-week period from 1 April to 14 April 2021. The study population comprised students less than 30 years of age enrolled in all majors (i.e., field of medical or life sciences, social sciences, and technology) at the University “Magna Græcia” of Catanzaro, which is located in the Southern part of Italy. A cut-off of 30 years was chosen in order to focus on young adults.

We hypothesized that the major could have an effect on the students’ knowledge, attitudes, and practices regarding antibiotic use, and to make sure that we have representatives from all the different majors, so we conducted a stratified sampling. Hence, we choose a random sample of 25 males and a random sample of 25 females from each of the 28 university’s majors. All students were recruited through the university email distribution list. The criteria of eligibility for the study were as follows: (i) age between 18 and 30 years and (ii) registered as an undergraduate student in the university. Students who declined to sign the informed consent were excluded from the study. An anonymous online survey was sent via institutional email to all the eligible students. On the first page of the online questionnaire, there was a personal data treatment information sheet at the end of which students could give their agreement in joining the study. An explanatory opening paragraph reporting the purpose of the study, advising that there was no obligation to complete the questionnaire, and reassuring that the information obtained would remain confidential, was also included. There were no identifiers linking students to the questionnaire, which was securely stored and transferred to a password-protected and encrypted computer. The questionnaire could only be submitted once for each electronic device in an effort to reduce potential repeat responses. A total of 1051 students responded to the survey.

### 4.2. Survey Instrument

The questionnaire was developed based on previous similar studies [30,33,34,35,36,37]. The questionnaire was pilot tested on a sample of 20 potential responders not included in the final sample. Minor refinements were made based on the feedback received from the responders before finalizing the questionnaire. The final survey was designed to be completed within 10 min and collected data on four core areas: (a) socio-demographic characteristics (closed-ended with multiple answers and open-ended questions); (b) knowledge about antibiotic use and ABR (7 questions with a “true/false” response format); (c) attitudes towards ABR and online purchase of pharmaceutical products and antibiotics (four items on a five-point Likert scale response format); (d) practices regarding antibiotic use and self-medication, use of the Internet to seek health information, and information about antibiotics and/or ABR and to buy pharmaceutical products (19 closed-ended questions with a multi-option response format). The questionnaire is available as Supplementary Material File S1.

The study was reviewed and approved by the Regional Human Research Ethics Committee (ID No. 104/2021/03/18).

### 4.3. Statistical Analysis

Statistical analysis was developed using STATA software program, version 16.1 [38]. Data were summarized using mean and standard deviations for continuous data and frequencies for categorical data. The inferential analysis was conducted in three stages. First, bivariate analyses were performed using the appropriate tests to examine the potential association between the outcomes of interest and the explanatory variables. Second, multiple logistic regression models were developed to investigate the relationship between the independent variables and the following outcomes of interest: self-medication with antibiotics (no = 0; yes = 1) (Model 1) and belief that ABR is a problem that could not be solved through the marketing of new antibiotics (no = 0; yes = 1) (Model 3). The selection of the variables was carried out by introducing knowledge-based possible confounders and the variables that in the bivariate analysis showed an association with the outcome of interest with a *p*-value lower than 0.25, as suggested by Hosmer et al. [39]. In the last stage of the analysis, the models were rebuilt to include the variables that in the previous analyses showed an association with the outcome of interest with a *p*-value lower than 0.05. An overall knowledge score was calculated by assigning one point for each correct answer and summing the scores of each statement (range 0–7). The overall knowledge score about antibiotics and ABR was analyzed through an ordinal regression model (Model 2), and the assumption of constancy of effects across categories was verified. The following variables were included in all models: gender (male = 0; female = 1), age (continuous), attending to medical or life sciences majors (no = 0; yes = 1), and the presence of chronic diseases (no = 0; yes = 1). In Models 1 and 3, the knowledge score about antibiotics and ABR (ordinal) was included. Furthermore, the variables belief that ABR is a problem that could not be solved through the marketing of new antibiotics (no =0; yes =1), Internet use to buy pharmaceutical products (no = 0; yes = 1), to seek health information (no = 0; yes = 1), and information about antibiotics and/or ABR (no = 0; yes = 1) were also included in Model 1. To avoid unstable estimates, the knowledge scores with an empty cell were removed from the model as a sensitivity analysis. Collinearity was explored using chi-square test for categorical variables and *t*-test or simple linear regression for continuous variables. Sensitivity analysis was performed by removing the collinear variables from each model. Goodness of fit tests were performed to assess the adequacy of logistic models. Adjusted OR and 95% CI were calculated.

The data set was deposited in Mendeley Data repository (doi:10.17632/94k3ds92y9.1).

## 5. Conclusions

The study findings highlighted gaps in knowledge, considerable antibiotic consumption, sometimes without prescription by a physician, together with an extensive Internet use to seek health-related information. Insights from the present study suggested that the challenge to public health practice is to facilitate the health-promoting use of the Internet in conjunction with health care providers to make young adults aware of concepts of rational use of antibiotics.

## Figures and Tables

**Table 1:** Distribution of practices regarding antibiotic consumption and Internet use according to general characteristics of the study population.

Statement	Correct Answer		Incorrect Answer		Gender				Age	Presence of Chronic Diseases			
					Male		Female			Yes		No	
	n	%	n	%	n	%	n	%	Mean ± SD	n	%	n	%
**I have taken antibiotics once in my life (1051)**	1022	97.2	29	2.8	309	95.4	713	98.1	22.8 ± 2.9	102	98.1	920	97.2
					χ^2^ = 6.1, 1 df, *p* = 0.013 *				t = −3.132, 1049 df, *p* = 0.002 *	χ^2^ = 0.3, 1 df, *p* = 0.583			
**I have taken antibiotics in the previous year (1022)**	640	62.6	382	37.4	178	57.6	462	64.8	22.7 ± 2.9	68	66.7	572	62.2
					χ^2^ = 4.8, 1 df, *p* = 0.029 *				t = −1.440, 1020 df, *p* = 0.150 *	χ^2^ = 0.8, 1 df, *p* = 0.374			
**I have self-medicated with antibiotics once in a lifetime (1022)**	251	24.6	771	75.4	77	24.9	174	24.4	23.2 ± 2.9	29	28.4	222	24.1
					χ^2^ = 0.03, 1 df, *p* = 0.861				t = −2.420, 1020 df, *p* = 0.016 *	χ^2^ = 0.9, 1 df, *p* = 0.338			
**Internet use to seek health information (1049)**	993	94.7	56	5.3	300	93.2	693	95.3	22.7 ± 2.9	95	91.4	898	95
					χ^2^ = 2.1, 1 df, *p* = 0.152				t = 1.053, 1047 df, *p* = 0.293	χ^2^ = 2.5, 1 df, *p* = 0.113			
**Internet use to seek information on antibiotics and/or ABR (1049)**	700	66.7	349	33.3	204	63.4	496	68.2	23 ± 2.9	76	73.1	624	66
					χ^2^ = 2.4, 1 df, *p* = 0.122				t = −2.629, 1047 df, *p* = 0.009 *	χ^2^ = 2.1, 1 df, *p* = 0.148			
**I have bought pharmaceutical products online (1049)**	179	17.1	870	82.9	59	18.3	120	16.5	23.6 ± 3	26	25	153	16.2
					χ^2^ = 0.5, 1 df, *p* = 0.471				t = −4.095, 1047 df, *p* < 0.001 *	χ^2^ = 5.1, 1 df, *p* = 0.023 *			
**I have bought antibiotics at online pharmacies (179)**	8	4.5	171	95.5	4	6.8	4	3.3	23.6 ± 2.7	2	4.5	6	3.9
					Fisher’s exact *p* = 0.294				t = 0.100, 177 df, *p* = 0.921	Fisher’s exact *p* = 0.390			

Number of respondents to the statements are in brackets. ABR = Antibiotic resistance, SD = Standard deviation. * *p*-value < 0.05 was considered as statistically significant.

**Table 2:** Results of the multiple and ordinal logistic regression models for potential determinants of the outcomes of interest.

**1. Multiple Logistic Regression Model. Outcome: Self-Medication with Antibiotics** **Log Likelihood = −549.754; Prob > Chi2 < 0.0001; Obs = 1021; Cox and Snell R-Square = 0.036; Nagelkerke R-Square = 0.053**			
**Variables**	**OR**	**95% CI**	***p***
**Internet use to buy pharmaceutical products**			
No *	1.00		
Yes	1.95	1.36–2.78	<0.001
**Internet use to seek information about antibiotics and/or ABR**			
No *	1.00		
Yes	1.69	1.20–2.38	0.002
**Age, continuous**	1.05	1.00–1.11	0.048
**Knowledge score** **about antibiotics and ABR** **, continuous**	0.91	0.81–1.01	0.089
**Internet use to seek health information**			
No *	1.00		
Yes	1.90	0.83–4.33	0.126
**Presence of chronic diseases**			
No *	1.00		
Yes	1.15	0.72–1.85	0.561
**Attending to medical or life sciences majors**			
No *	1.00		
Yes	0.93	0.67–1.28	0.659
**Gender**			
Male *	1.00		
Female	0.97	0.70–1.35	0.877
**Belief that ABR is a problem that could not be solved through the marketing of new antibiotics**			
No *	1.00		
Yes	1.02	0.75–1.39	0.901
**2. Ordinal Regression Model. Outcome: Knowledge about Antibiotics and ABR** **Log Likelihood = −1641.989; Prob > Chi2** **< 0.0001** **; Obs = 1028; Cox and Snell R-Square = 0.120; Nagelkerke R-Square = 0.125**			
**Variables**	**Coefficient**	**95% CI**	***p***
**Age, continuous**	0.09	0.05–0.12	<0.001
**Attending to medical or life sciences majors**			
No *	1.00		
Yes	1.13	0.90–1.37	<0.001
**Gender**			
Male *	1.00		
Female	−0.25	−0.50–0.01	0.043
**Presence of chronic diseases**			
No *	1.00		
Yes	0.08	−0.28–0.44	0.671
**3. Logistic Regression Model. Outcome: Belief that ABR is a Problem that Could not be Solved through the Marketing of New Antibiotics** **Log Likelihood = −654.660; Prob > Chi2** **< 0.0001** **; Obs = 1051; Cox and Snell R-Square = 0. 700; Nagelkerke R-Square = 0.095**			
**Variables**	**OR**	**95% CI**	***p***
**Knowledge score about antibiotics and ABR,** **continuous**	1.21	1.09–1.33	<0.001
**Attending to medical or life sciences majors**			
No *	1.00		
Yes	2.02	1.52–2.69	<0.001
**Age, continuous**	1.06	1.02–1.11	0.007
**Presence of chronic diseases**			
No *	1.00		
Yes	1.74	1.14–2.65	0.011
**Gender**			
Male *	1.00		
Female	1.15	0.86–1.53	0.342

* Reference category. ABR = Antibiotic resistance; Log likelihood = log-likelihood function; Obs = observations; Prob = probability; chi2 = chi-square, OR = Odds Ratio; 95% CI = 95% confidence interval; *p* = *p*-value.

**Table 3:** Distribution of knowledge about antibiotic and ABR according to general characteristics of the study population.

Statement	Correct Answer		Incorrect Answer		Gender			Age		Presence of Chronic Diseases			
					Male		Female			Yes		No	
	n	%	n	%	n	%	n	%	Mean ± SD	n	%	n	%
**Antibiotics are not useful to treat viral infections**	649	61.8	402	38.3	217	67	432	59.4	23.3 ± 2.9	66	63.5	583	61.6
					χ^2^ = 5.4, 1 df, *p* = 0.020 *			t = −7.271, 1049 df, *p* < 0.001 *		χ^2^ = 0.1, 1 df, *p* = 0.705			
**You cannot stop taking the antibiotic when you start feeling better**	912	86.8	139	13.2	273	84.3	639	87.9	23 ± 2.9	100	96.2	812	85.7
					χ^2^ = 2.6, 1 df, *p* = 0.108			t = −5.167, 1049 df, *p* <0.001 *		χ^2^ = 8.8, 1 df, *p* = 0.003 *			
**You cannot take antibiotics that individuals with the same symptoms have used without consulting your doctor**	1029	97.9	22	2.1	308	95.1	721	99.2	22.8 ± 2.9	103	99	926	97.8
					χ^2^ = 18.5, 1 df, *p* < 0.001 *			t = 0.179, 1049 df, *p* = 0.858		χ^2^ = 0.7, 1 df, *p* = 0.396			
**Self-medication with antibiotics contributes to the spread of ABR**	815	77.5	236	22.5	263	81.2	552	75.9	23.1 ± 2.9	88	84.6	727	76.8
					χ^2^ = 3.5, 1 df, *p* = 0.060			t = −6.116, 1049 df, *p* < 0.001 *		χ^2^ = 3.3, 1 df, *p* = 0.069			
**You cannot legally buy antibiotics without a prescription at the community pharmacy**	538	51.2	513	48.8	160	49.4	378	52	22.8 ± 2.9	54	51.9	484	51.1
					χ^2^ = 0.6, 1 df, *p* = 0.434			t = −0.212, 1049 df, *p* = 0.832		χ^2^ = 0.0, 1 df, *p* = 0.875			
**You cannot legally buy antibiotics without a prescription at the online pharmacy**	393	37.4	658	62.6	130	40.1	263	36.2	23 ± 2.9	34	32.7	359	37.9
					χ^2^ = 1.5, 1 df, *p* = 0.222			t = −1.709, 1049 df, *p* = 0.088		χ^2^ = 1, 1 df, *p* = 0.297			
**Online pharmacies could sell pharmaceutical products of poor quality**	391	37.2	660	62.8	148	45.7	243	33.4	22.7 ± 3	38	36.5	353	37.3
					χ^2^ = 14.4, 1 df, *p* < 0.001 *			t = 0.713, 1049 df, *p* = 0.476		χ^2^ = 0.0, 1 df, *p* = 0.883			

ABR = Antibiotic resistance; SD = standard Deviation. * *p*-value < 0.05 was considered as statistically significant.

## Data Availability

The data presented in this study are openly available in Mendeley Data repository (doi:10.17632/94k3ds92y9.1).

## Supplementary Materials

The following are available online at https://www.mdpi.com/article/10.3390/antibiotics10091091/s1, File S1: Questionnaire.
