# Supplementary material for: The Internet: Friend or Foe of Antibiotic Resistance? Results of a Cross-Sectional Study among Italian University Students
Source: Antibiotics (Basel). 2021 Sep 9;10(9):1091. doi: 10.3390/antibiotics10091091 (PMC8470861; doi:10.3390/antibiotics10091091)
Supplement: Supplementary file 1 [file antibiotics-10-01091-s001.zip › antibiotics-1350724-supplementary.pdf]

UNIVERSITY OF CATANZARO
“MAGNA GRÆCIA”
MEDICAL SCHOOL OF HEALTH SCIENCES

The Internet: friend or foe of antibiotic resistance? Results of a cross-sectional study among Italian university students

QUESTIONNAIRE

The purposes of the study were to investigate knowledge and attitudes towards antibiotics and antibiotic resistance (ABR), and to assess the extent of practices regarding antibiotic consumption and Internet use.
According to General Data Protection Regulation (EU) 2016/679, data will be analyzed only in an aggregate way and there will be no nominal data spreading. Precautions have been taken in order to guarantee confidentiality of gathered data and anonymity of respondents, so you can feel completely free to respond.

A. SOCIO-DEMOGRAPHIC CHARACTERISTICS

This section is designed to gather information about your socio-demographic characteristics.

A.1. How old were you on your last birthday? ________

A.2. What is your gender? ☐ Male ☐ Female

A.3. What is your nationality? ☐ Italian ☐ Other (specify) __________________

A.4. What course do you attend at university? ______

A.5. Do you have any chronic diseases? ☐ Yes ☐ No

A.6 How often do you use the Internet? ☐ Daily ☐ Every week ☐ Every month ☐ Less than once a month ☐ When necessary

B. KNOWLEDGE

This section is designed to gather information about knowledge regarding antibiotics and ABR. Please indicate the answer which best describes your understanding.

**B.1.**	Antibiotics are effective for viral infections	☐True	☐False
**B.2.**	You can stop taking the antibiotic when you start feeling better	☐True	☐False
**B.3**	You can take antibiotics that individuals with the same symptoms have used without consulting your doctor	☐True	☐False
**B.4.**	Self-medication with antibiotics contributes to the spread of ABR	☐True	☐False
**B.5.**	You can legally buy antibiotics without a prescription at the community pharmacy	☐True	☐False
**B.6.**	You can legally buy antibiotics without a prescription at the online pharmacy	☐True	☐False
**B.7.**	Online pharmacies could sell pharmaceutical products of poor quality	☐True	☐False

C. <u>ATTITUDES</u>

This section is designed to gather information about attitudes towards ABR and online purchase of pharmaceutical products and antibiotics. Please indicate the answer which best describes your belief.

**C.1.**	ABR is a problem that could be solved through the marketing of new antibiotics	☐Strongly agree	☐Agree	☐I do not know	☐Disagree	☐Strongly disagree
**C.2.**	The online purchase of pharmaceutical products is safe	☐Strongly agree	☐Agree	☐I do not know	☐Disagree	☐Strongly disagree
**C.3.**	The online purchase of pharmaceutical products could be a negative possibility due to an increased risk of receiving counterfeit substances	☐Strongly agree	☐Agree	☐I do not know	☐Disagree	☐Strongly disagree
**C.4.**	The online purchase of antibiotics could be a negative possibility due to an increased risk of receiving counterfeit substances	☐Strongly agree	☐Agree	☐I do not know	☐Disagree	☐Strongly disagree

D. <u>PRACTICES</u>

Below are some questions about your personal experiences regarding antibiotics and ABR. Please indicate the answer which best describes your practices.

D.1. Have you taken an antibiotic once in your life?

☐Yes ☐No (Skip to D.7.) ☐I do not remember (Skip to D.7.)

D.2. Have you taken an antibiotic in the previous 12 months?

☐Yes ☐No (Skip to D.7.) ☐I do not remember (Skip to D.7.)

D.3. Have you ever self-medicated with antibiotics at least once in your life?

☐Yes ☐No (Skip to D.7.) ☐I do not remember (Skip to D.7.)

D.4. Why did you self-medicate with antibiotics?

☐I did not have time to consult the physician ☐The physician did not want to prescribe it

☐Other (specify) ________________

D.5. Have you ever had difficulty in getting an antibiotic from a pharmacist without a prescription?

☐Yes ☐No ☐I do not remember

D.6. Do you keep leftover antibiotics at home for future use?

☐Yes ☐ No, I return unused antibiotics to pharmacies ☐ No, specify the reason, please________________________

D.7. Have you ever used the Internet to search for health information?

☐Yes ☐No (Skip to D.10.) ☐I do not remember (Skip to D.10.)

D.8. How often have you used the Internet to search for health information?

☐Daily ☐Every week ☐Every month ☐Less than once a month ☐When necessary

D.9. What are the reasons you search the Internet for health information? (It is possible to indicate more than one answer)

☐To learn more about a disease ☐To manage my health more independently

☐To find out more about my and/or of my family’s/friend’s health status ☐The information provided by the doctor was insufficient

☐I was not comfortable asking my physician for more information ☐ To learn more about a pharmacological treatment/disease treatment

☐Other (specify other reason) ___________

D.10. Have you ever used the Internet to seek information on antibiotics and/or ABR?

☐Yes ☐No ☐I do not remember

D.11. Have you ever bought pharmaceutical products online?

☐Yes ☐No ☐I do not remember

D.12. For what reason (s) did you buy pharmaceutical products online? (It is possible to indicate more than one answer)

☐Inability to go to the pharmacy ☐ It is cheaper than the community pharmacy

☐ It is more convenient than the community pharmacy ☐ Other (specify other reason) ________________

D.13. Have you ever bought antibiotics online?

☐Yes ☐No ☐I do not remember

The questionnaire is finished, we thank you for your valuable collaboration!
